# Effect of Xylitol and Fluoride Varnish on Biofilm and Saliva in Orthodontic Patients: A Triple‐Blind Randomized Clinical Trial

**DOI:** 10.1002/cre2.70062

**Published:** 2025-02-23

**Authors:** Neda Babanouri, Sarina Sahmeddini, Setayesh Khadang, Abdollah Bazargani

**Affiliations:** ^1^ Orthodontic Research Center, School of Dentistry Shiraz University of Medical Sciences Shiraz Iran; ^2^ Department of Bacteriology and Virology, School of Medicine Shiraz University of Medical Sciences Shiraz Iran

**Keywords:** fluorides, orthodontic appliances, *Streptococcus mutans*, Xylitol

## Abstract

**Objectives:**

Fixed orthodontic appliances are associated with higher levels of plaque and saliva bacteria, which contribute to dental caries. The effects of combining xylitol and fluoride, both used in caries prevention, are uncertain. Thus, this study assessed the combined impact of fluoride and xylitol varnish on bacteria in saliva and biofilms around orthodontic brackets.

**Materials and Methods:**

A single‐center, four‐arm, parallel‐group, triple‐blind, randomized clinical trial was conducted. A total of 120 patients who required fixed orthodontic treatment were included. Patients were sorted into one of the following groups at random: fluoride, xylitol, combined fluoride and xylitol, and control. Biofilm and saliva sampling was performed at two intervals: T0 (first session of bracket bonding, before application of the varnish) and T1 (6 weeks after application of the varnish). The number of *Streptococcus mutans* and *Lactobacillus* was counted using the CFU method.

**Results:**

The relative number of *S. mutans* and *lactobacilli* in saliva and biofilm significantly decreased following the application of fluoride and combined fluoride/xylitol varnish (*p* < 0.05) and were more effective than xylitol varnish. There was no significant difference between fluoride and combined fluoride/xylitol varnishes regarding changes in *S. mutans* and *Lactobacillus* colonies in saliva and dental biofilms. Additionally, there was no significant difference between xylitol and the two other active varnishes regarding reduction in biofilm *S. mutans* and *Lactobacillus*.

**Conclusion:**

There was no significant difference between fluoride and combined fluoride/xylitol varnishes regarding changes in the studied bacteria in saliva and dental biofilms, and they were more effective than xylitol varnish.

**Trial Registration:**

The Iranian Registry of Clinical Trial identifier: IRCT20181121041713N4; https://fa.irct.ir/trial/58543

## 1 Introduction

Fixed orthodontic treatment is commonly used to correct malocclusions and achieve optimal occlusions. However, it can also increase the risk of tooth decay due to plaque accumulation and food debris around the brackets and wires. Studies have shown that fixed orthodontic appliances can significantly increase the prevalence of dental caries (Perkowski et al. 2019; Kozak et al. 2021; Enerbäck et al. 2019). Studies have reported a 16%–39% increase in the incidence of dental caries during orthodontic treatment compared to that before treatment (Enerbäck et al. 2023; Salerno et al. 2024).

The acidic environment created by bacterial fermentation of dietary carbohydrates in dental plaque can cause demineralization of the tooth enamel and subsequent cavity formation. *Streptococcus mutans* and *Lactobacillus* species are known to be the most significant contributors to dental caries (Najafi et al. 2020; Moshaverinia et al. 2022). Therefore, controlling the levels of *S. mutans* and *Lactobacillus* in the oral cavity is crucial for preventing dental caries, which can be achieved through measures such as maintaining good oral hygiene practices, reducing sugar consumption, and using antimicrobial agents.

As a preventive measure, orthodontists may prescribe fluoride varnish to promote remineralization and prevent enamel demineralization. In addition, xylitol, a natural sugar substitute, inhibits the growth of cariogenic bacteria and promotes the production of saliva, which can buffer the pH of the oral environment (Silva et al. 2021).

The combination of fluoride and xylitol varnish has been suggested as a more effective method for preventing caries (Biradar et al. 2020). Cardoso et al. (2014) reported that xylitol–fluoride formulations significantly enhanced enamel remineralization. However, a pH‐cycling study on bovine teeth found no significant benefit from adding xylitol to fluoride varnish (Vongsavan et al. 2014). Similarly, Zajkani et al. (2018) concluded that fluoride mouthwash alone was more effective than a fluoride–xylitol combination in inhibiting *S. mutans* and *L. acidophilus* growth. These mixed results highlight the need for further research.

The rationale for conducting this study was based on the need for effective preventive measures against dental caries during orthodontic treatment. This study aimed to address this gap in knowledge by evaluating the efficacy of a combination of xylitol and fluoride varnish in reducing biofilm formation and levels of cariogenic bacteria in fixed orthodontic patients through a clinical trial. The results of this study may inform clinical practice by identifying effective preventive measures to reduce the risk of caries during orthodontic treatment.

## 2 Materials and Methods

### 2.1 Trial Design

The present study was a single‐center, triple‐blind, four‐arm, parallel‐group randomized clinical trial design with a 1:1:1:1 allocation ratio, following CONSORT guidelines. The methods remained unchanged after trial commencement.

### 2.2 Participants, Eligibility Criteria, and Settings

Patients scheduled for comprehensive orthodontic treatment at the Orthodontic Department, School of Dentistry, Shiraz University of Medical Sciences, Shiraz, Iran, were invited to participate in the study. The trial was approved by the Ethics Committee of Shiraz University of Medical Sciences (code: IR.SUMS.DENTAl.REC.1400.036) and registered at the Iranian Registry of Clinical Trials (IRCT20181121041713N4).

Patients between the age group of 12–30 years with permanent dentition who needed fixed orthodontic treatment were included in the trial. Participants also had to have good oral hygiene (plaque index of < 20%) and without any visible caries or tooth abnormalities (fluorosis, hypocalcification, or developmental defects). If a patient displayed any of the following, they were excluded: active gingivitis and periodontitis, systemic diseases, pregnancy, usage of medications that would likely affect dental biofilm, severe crowding of anterior teeth, and history of smoking and mouth breathing. All participants were asked to read and sign an informed consent form that clearly explained the aim of the study and probable risks and benefits.

### 2.3 Sample Size Calculation

The sample size for this trial was calculated based on the pilot trial involving 10 *S. mutans* biofilm samples for a power of 80%, alpha error at 5% with mean and standard deviation of 1.89 ± 0.82 and 1.25 ± 0.81 before and after varnish application, respectively, using the following formula:

$$N=\frac{{2(r+1)(z_{\alpha }+z_{1-\beta })}^{2}\sigma^{2}}{rd^{2}}$$

The primary outcome measure was the number of *S. mutans* bacteria (colony‐forming units per milliliter, CFU/mL). A minimum of 28 participants per group was required. To account for potential dropouts, the sample size was increased to 30 participants per group.

### 2.4 Randomization and Allocation Concealment

The web program RANDOM.ORG was used to randomly assign patients in a 1:1:1:1 ratio to the four experimental groups using the block randomization method using an 8‐block size. Subsequently, the cards were shuffled and concealed in opaque envelopes to increase the randomness of the allocation sequence. Each patient had to select a sealed envelope to assign the xylitol, fluoride, combination varnish, or control group. Allocation concealment was performed to avoid selection bias. A nursing assistant who was not involved in the trial was in charge of patient enrollment and randomization.

### 2.5 Blinding

All varnishes were the same color and spilled into identical tubes that were color‐coded by a person not involved in this study; therefore, the clinician, laboratory technician, patients, and statistician were all blinded to the treatment groups.

### 2.6 Intervention

After being screened for eligibility, 150 patients were analyzed, and 120 of them met the requirements for inclusion. Four groups of patients were chosen at random, and they received one of the following varnishes. The base of varnishes was prepared by blending fully hydrogenated rosin and absolute ethanol in a 3:1 (w/w) ratio. This mixture was stirred in a closed container at room temperature for 24 h, and then the particles were added.

1. Fluoride group: dental varnish with 5% NaF.
2. Xylitol group: dental varnish with 10% xylitol.
3. Combined group: dental varnish contained 5% NaF+10% xylitol.
4. Control group: dental varnish without NaF and xylitol.

Before bonding, the patient's teeth were debrided and polished, and then they underwent orthodontic treatment provided by the same orthodontist using fixed pre‐adjusted edgewise appliances (Mini Master Bracket, 0.022‐in MBT prescription; American Orthodontics, USA). A standardized bonding method was applied following the directions provided by the manufacturer.

All patients were given toothpaste (1450 ppm F, Colgate Total Advanced; Colgate–Palmolive, New York, NY, USA) and a soft‐bristled adult brush (Trisa Bracket Clean; Trisa AG, Triengen, Switzerland) at the end of the bonding session. They were instructed to brush their teeth for 2 min in the morning and at night using an up‐and‐down motion on their front teeth and a circular motion on their back teeth. Throughout the trial, they also abstained from using mouthwash, chewing gum, and antibiotics. No further fluoride supplements were given throughout the investigation. Home care compliance was assessed based on self‐reported data from participants. While acknowledging the inherent limitations of self‐reported measures, we relied on the participants' honesty and commitment to the study protocol.

Before varnish was applied, the teeth were dried with compressed air and isolated after the mouth was cleaned with water, and the plaque was removed with a toothbrush without toothpaste. Before the application of the varnish, the same oral prophylaxis was administered for the teeth at every visit. The operator then applied two drops of a specified varnish around the brackets to all maxillary and mandibular teeth using a single‐tufted brush with a dome‐shaped brush head and gauze sponges (2 × 2) for each patient. 1 min was given for the varnish to dry. All varnishes used in this study were obtained from Asia ChemiTeb Co. (Tehran, Iran). The patients were told to abstain from food and liquids for 2 h and to wait until the next day to wash their teeth. After 6 weeks, the process was repeated.

### 2.7 Biofilm and Saliva Sample Collection Method

Biofilm and saliva samples were collected at the beginning of the study, just before the application of the varnish on their first session of bracket bonding (T0), and 6 weeks after the first varnish application (T1). The participants were instructed to spit 4 mL into sterile Falcon tubes filled with 1 mL of normal saline solution. Each participant was instructed to abstain from food and liquids for 1 h before sampling. The microbiology division of the medical college associated with Shiraz University of Medical Sciences received the samples that had been gathered.

After using cotton rolls to isolate the labial maxillary surfaces, supragingival biofilm samples were obtained from all maxillary teeth with bracket labial and buccal surfaces. The samples were taken from the cervical region using the tip of a sterile, triangular wooden toothpick. For each quadrant, a toothpick was utilized. The two tips with adhering biofilms were removed and transferred to one bottle with 1.0 mL of double distilled deionized water and sent to the microbiology department.

### 2.8 Sample Preparation and Analysis

The supernatant was removed after centrifuging saliva samples at 12,500 rpm for 10 min. A concentrated sample suspension was made by suspending the precipitate in 1 mL of phosphate‐buffered saline. A single loop of the concentrated suspension was used to inoculate the MSB agar (*S. mutans*) and MRS (*lactobacilli*) using the standard streak plate method. The MSB plates were incubated in a candle jar at 37°C for 24 h, and MRS plates were incubated without a candle jar at the same time. The number of colony‐forming units of *S. mutans* and *Lactobacillus* was counted and identified based on their characteristic colony morphology.

To cultivate bacteria, pooled plaque samples were placed into 2 mL Eppendorf tubes with BHI broth. The samples were vortexed for 5 min to homogenize them, and 1 mL of each sample was diluted from 1:10 to 1:106. Following dilution, 0.1 mL of the samples were taken and placed on agar plates to test for microbial growth. Under aerobic conditions with 5% carbon dioxide supplementation, the agar plates were incubated at 37°C for 48 h. Bacterial colonies were counted and identified based on their characteristic colony morphology.

### 2.9 Statistical Analysis

The Statistical Package for Social Sciences (version 15.0, SPSS Inc., Chicago, IL, USA) was used to analyze the data. To evaluate the homogeneity of the groups in terms of age and sex, one‐way ANOVA and Chi‐square tests were used. Given the baseline variability in *S. mutans* and *lactobacilli* counts among groups, the changes from baseline to T1 were calculated for each parameter and used in the statistical analysis to account for initial disparities.

Nonparametric tests were chosen due to the non‐normal distribution of the data and the presence of zero values in certain groups (e.g., *lactobacilli* in the xylitol group). Since nonparametric methods are based on ranks rather than raw values, they are robust against the influence of extreme values and handle distributions where zero is the mode rather than the mean. The following statistical tests were applied: The Wilcoxon signed‐rank test for intra‐group comparisons (T1 vs. T0), the Kruskal–Wallis test for differences in baseline‐adjusted changes among all study groups, the Mann–Whitney *U* test for pairwise comparisons of baseline‐adjusted changes between groups. Statistical significance was set at *p* < 0.05.

## 3 Results

### 3.1 Participant Flow

**Figure 1:**
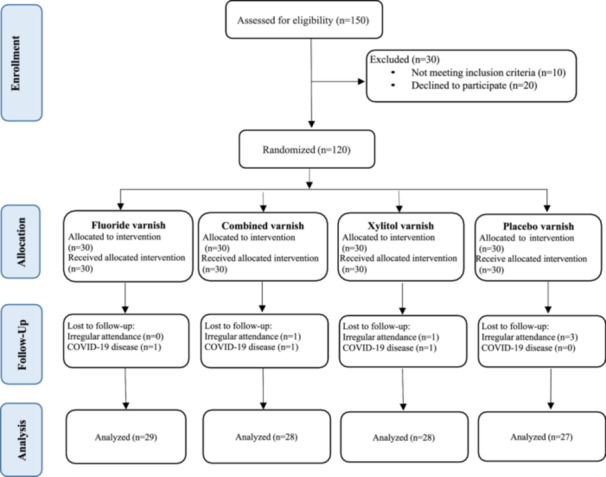
CONSORT flow diagram showing the progress of all the participants throughout the trial.

A total of 120 patients were enrolled in this study from October 2021 to January 2022; however, eight patients were excluded from the trial as a result of their irregular attendance or COVID‐19 disease. The final sample size consisted of 112 patients in the four groups. Figure 1 shows a CONSORT flow diagram of the enrollment, intervention distribution, follow‐up, and data analysis procedures.

### 3.2 Baseline Data

Table 1 details the participant demographics, including age and gender information. The distribution of patients between the groups was not significantly different in terms of age or gender (*p* = 0.210 and *p* = 0.119, respectively), suggesting that the patients were properly randomized. Additionally, there was no difference in the length of orthodontic treatment between the four groups (mean: 18.7±3.5, *p* = 0.80).

**Table 1:** Demographic characteristics of four study groups.

		Fluoride	Fluoride + xylitol	Xylitol	Placebo
Gender (*n*, %)	Male	7, 24.1%	3, 10.7%	5, 17.9%	9, 33.3%
	Female	22, 76.9%	25, 89.3%	23, 82.1%	18, 66.7%
Age (year)	Mean ± SD (Max/min)	20.34 ± 5.4 (12/30)	18.54 ± 4.45 (13/29)	17.50 ± 4.1 (12/27)	19.41 ± 4.1 (12/30)

*Note:* No significant differences were found between the groups regarding demographic variables (*p* > 0.05).

### 3.3 Outcome

Samples of 112 patients were finally analyzed before and after the varnish applications and are presented in Table 2. According to the results, fluoride varnish and the combination of fluoride and xylitol varnish significantly decreased *S. mutans* and *lactobacilli* in both saliva samples and *S. mutans* biofilm samples (*p* < 0.05). In contrast, xylitol only reduced the biofilm of *S. mutans*, and in other samples, there was an increase in the number of bacteria, although the difference was not significant (*p* > 0.05). Generally, none of the treatments significantly reduced the biofilm *lactobacilli*. In the control group that received a placebo, bacterial growth increased in all groups; however, the increase in salivary bacterial growth was not significant (*p* > 0.05).

**Table 2:** Intra‐group comparisons of bacterial growth based on the Wilcoxon signed‐rank test.

		T0	T1	
Parameters	Group	Median	Median	*p* value
SL	Fluoride	2.25	1.20	0.010*
	Fluoride + xylitol	1.74	0.15	0.001*
	Xylitol	1.88	1.52	0.424
	Control	1.25	1.69	0.005
BL	Fluoride	1.00	0.00	0.053
	Fluoride + xylitol	0.00	0.00	0.209
	Xylitol	0.30	0.84	0.568
	Control	0.00	1.07	0.000*
SS	Fluoride	1.81	1.34	0.013*
	Fluoride + xylitol	1.86	0.92	0.010*
	Xylitol	1.58	1.64	0.866
	Control	2.05	2.08	0.471
BS	Fluoride	1.72	0.90	0.004*
	Fluoride + xylitol	2.00	1.17	0.000*
	Xylitol	0.84	1.20	0.200
	Control	1.41	1.56	0.0212

* Significant results (*p* < 0.05); SL: *lactobacilli* in saliva samples; BL: *lactobacilli* in biofilm samples; SS: *Streptococcus mutans* in saliva sample; BS: *Streptococcus mutans* in biofilm samples; T0: At the beginning of the study, just before the application of the varnish; T1: Six weeks after the first varnish application.

The baseline distribution of biofilm *S. mutans* (BS0), *lactobacilli* in the microbial biofilm (BL0), and salivary *lactobacilli* colonies (SL0) was not uniform across the study groups. Some groups exhibited zero counts at baseline, with zero being the mode for certain parameters.

To account for these disparities, the changes from baseline to T1 (Diff‐SS, Diff‐SL, Diff‐BS, Diff‐BL) were calculated and analyzed. Using nonparametric methods ensured that the analyses were robust to these baseline differences, as they are rank‐based and not affected by the raw magnitude of extreme or zero values. The Kruskal–Wallis test identified statistically significant differences in baseline‐adjusted changes among groups (Diff‐SS, Diff‐SL, Diff‐BS, Diff‐BL; *p* < 0.05; *p* < 0.05; *p* < 0.05; Table 3). Pairwise comparisons using the Mann–Whitney *U* test are shown in Table 4. Table 3 shows that there were statistically significant differences in Diff‐SS, Diff‐SL, Diff‐BS, and Diff‐BL among the four study groups (*p* < 0.05).

**Table 3:** Differences of all parameters at T1 and T0 among total data based on Kruskal–Wallis test.

Parameters	Group	*P* value
Diff‐BS	All groups	0.000*
Diff‐BL	All groups	0.000*
Diff‐SS	All groups	0.003*
Diff‐SL	All groups	0.000*

* Significant differences were found between the data (*p* < 0.05). Diff‐BS: Differences in *Streptococcus mutans* colony count in biofilm samples at T1 and T0 (BS1–BS0); Diff‐BL: Difference in *lactobacilli* colony count in biofilm samples at T1 and T0 (BL1–BL0); Diff‐SL: Difference in *lactobacilli* colony count in saliva samples at T1 and T0 (SL1–SL0); Diff‐SS: Difference in *Streptococcus mutans* colony count in saliva samples at T1 and T0 (SS1–SS0).

**Table 4:** Comparison of changes between groups based on the Mann–Whitney test.

Sample 1–sample 2	Parameters	*p* value
Fluoride – fluoride + xylitol	Diff‐BS	0.975
	Diff‐BL	0.050
	Diff‐SL	0.867
	Diff‐SS	0.587
Fluoride – xylitol	Diff‐BS	0.083
	Diff‐BL	0.115
	Diff‐SL	0.002*
	Diff‐SS	0.036*
Fluoride + xylitol − xylitol	Diff‐BS	0.131
	Diff‐BL	0.565
	Diff‐SL	0.000*
	Diff‐SS	0.035*
Fluoride − control	Diff‐BS	0.000*
	Diff‐BL	0.000*
	Diff‐SL	0.001*
	Diff‐SS	0.001*
Fluoride + xylitol – control	Diff‐BS	0.000*
	Diff‐BL	0.003*
	Diff‐SL	0.000*
	Diff‐SS	0.006*
Xylitol – control	Diff‐BS	0.005*
	Diff‐BL	0.002*
	Diff‐SL	0.833
	Diff‐SS	0.381

* Significant differences were found between the data (*p* < 0.05). Diff‐BS: Differences in *Streptococcus mutans* colony count in biofilm samples at T1 and T0 (BS1–BS0); Diff‐BL: Differences in *lactobacilli* colony count in biofilm samples at T1 and T0 (BL1–BL0); Diff‐SL: Differences in *lactobacilli* colony counts in saliva samples at T1 and T0 (SL1–SL0); Diff‐SS: Differences in *Streptococcus mutans* colony counts in saliva samples at T1 and T0 (SS1–SS0).

The results of the Mann–Whitney test showed that there was no significant difference between fluoride and combined fluoride/xylitol varnishes regarding changes in *S. mutans* and *Lactobacillus* colonies in the saliva and dental biofilm (Table 4). Additionally, there was no significant difference between xylitol and the other two active varnishes for Diff‐BS and Diff‐BL.

Nevertheless, both fluoride and combined varnishes were more effective in reducing *S. mutans* and *Lactobacillus* colonies in saliva than the placebo, as there was a significant difference between these active varnishes and control varnishes (*p* < 0.05). Moreover, there were significant differences between xylitol and other varnishes in the reduction of salivary *S. mutans* and *Lactobacillus* (Table 4).

## 4 Discussion

Given the widespread use of orthodontic treatments, clinicians and researchers are increasingly interested in minimizing side effects, particularly dental caries. To this end, we conducted a clinical trial to evaluate the efficacy of various treatments, namely fluoride, xylitol, and a combination of xylitol and fluoride varnish, to mitigate the growth of *S. mutans* and *lactobacilli* in both saliva and biofilm, both of which are commonly suspected of contributing to tooth decay.

The findings of this study revealed a significant decrease in the relative number of *S. mutans* and *lactobacilli* in both saliva and biofilm after the application of fluoride and the combined fluoride/xylitol varnish. This outcome can be attributed to the antimicrobial action of fluoride, which targets three main aspects: acidogenicity, endurance, and adherence to the tooth surface (Liao et al. 2017). In contrast, xylitol reduces microbial load through several mechanisms, including anti‐adhesion, oxidative stress, low permeability, and futile metabolism (Janakiram et al. 2017).

Several studies have explored the effectiveness of xylitol‐based products in reducing dental caries in humans. Antonio et al. (2011) conducted a systematic review and found that xylitol‐based candies and lozenges significantly reduced caries incidence by inhibiting *S. mutans* growth and promoting remineralization. Marghalani et al. (2017) demonstrated that xylitol is particularly effective in pediatric populations for reducing caries, suggesting its potential as an adjunctive agent in caries prevention strategies.

Nevertheless, there was no significant difference between the fluoride and combined fluoride/xylitol varnishes regarding the changes. This outcome is in line with an in vitro investigation by Soleymani et al. (2021), who used the disk diffusion method to examine the antibacterial properties of three popular fluoride varnish types against two cariogenic bacteria, *S. mutans* and *Lactobacillus acidophilus*. They found that each varnish had the best antibacterial effect against both bacteria; however, Polimo (xylitol + fluoride) and FluoroDose (fluoride) varnishes had comparable antimicrobial properties.

However, Maehara et al. found that combining fluoride (0.6 Mm) and xylitol (60 mM) inhibited acid formation more effectively than fluoride or xylitol alone. They tested the combined inhibitory impact of fluoride and xylitol on acid generation by *S. mutans* and *S. sobrinus*. They concluded that the proportion of lactic acid in the overall amount of acidic end products was reduced by adding fluoride and xylitol. In contrast, the proportion of formic and acetic acids increased. They found that fluoride inhibited the bottom portion of the glycolytic pathway, whereas xylitol inhibited the upper part of the process based on their analysis of intracellular glycolytic intermediates (Maehara et al. 2005). Similarly, another study evaluated the level of salivary *S. mutans* after rinsing with xylitol, fluoride, and a combination of xylitol and fluoride solutions compared with distilled water. The participants were children with high levels of *S. mutans* (> 105 CFU/mL) who were equally divided into 10 mL of 0.05% (w/v) sodium fluoride (NaF), 12.5% (w/v) xylitol, or 0.05% (w/v) NaF + 12.5% (w/v) xylitol three times daily for 1 min over 10 weeks. To measure the number of salivary bacteria, baseline, 5 and 10‐week paraffin‐stimulated whole saliva samples were taken. Within 5 weeks, after 10 weeks, and after 10 weeks relative to baseline, they saw significant drops in *S. mutans* in participants receiving 0.05% NaF + 12.5% xylitol over other groups and 12.5% xylitol alone (Arunakul et al. 2011).

The variance in the varnish formulation may be the reason for the discrepant findings. Previous research, such as that conducted by Shen et al., has shown that the maintenance and release behavior of different fluoride varnishes can be influenced not only by dosage but also by differences in carriers and the degree of uniformity. They noted that even when doses are taken from the same tube, the fluoride content can differ, and the nonuniform appearance of the varnish when it is dispensed indicates heterogeneity in the ingredients, leading to variable fluoride content across different varnishes. This, in turn, can impact the effectiveness of the antibacterial effect (Shen and Autio‐Gold 2002).

Moreover, variations in *S. mutans* adhesion types and human genetic factors significantly influence caries outcomes. Sheng et al. (2024) identified that human PRH1 and PRH2 genetic profiles determine susceptibility or resistance to *S. mutans* colonization, thereby specifying different microbial profiles and influencing caries risk. These findings underscore the importance of considering genetic predisposition and microbial diversity when designing caries prevention strategies. Future research could explore these interactions further to personalize preventive treatments.

Additionally, the current study found that using fluoride alone or combined with xylitol is more effective in reducing the number of bacteria in saliva than in xylitol. This may be because the appropriate amount of xylitol for preventing bacteria has not yet been determined; however, chewing gum is an excellent way to deliver it. Therefore, the addition of fluoride to xylitol as a varnish may be beneficial. This conclusion is consistent with a previous study conducted on 80 healthy children aged 8–9 years old, who rinsed their mouths with fluoride, xylitol, or a combination of both three times a day for 10 weeks. The inhibitory effect of xylitol alone was lower than that of xylitol combined with fluoride in the form of a mouthwash (Arunakul et al. 2011). Stecksén‐Blicks et al. (2004) also noted that although *S. mutans* strains changed after regular daily low‐dose xylitol use, the long‐term total bacteria count in plaque and saliva, as well as plaque acidogenicity remained unaltered.

In the control group, which received a placebo varnish, there was an increase in bacterial growth, including *lactobacilli* levels in biofilm samples, over the study period, though this was not statistically significant. This increase aligns with the hypothesis that orthodontic appliances may create an environment conducive to bacterial growth. However, the three active varnishes—fluoride, fluoride + xylitol, and xylitol alone—were effective at maintaining stable *lactobacilli* levels in biofilm samples, suggesting that they may help counteract this tendency in patients undergoing orthodontic treatment. Although these varnishes did not significantly reduce *lactobacilli* levels, their ability to prevent further increases implies a protective effect.

The limitations of this trial include assessing only patients with good oral hygiene, a predominantly female sample from a single institution, and a short study duration. We recommend longer clinical trials to better monitor potential side effects or adverse outcomes during the study.

## 5 Conclusion

The study found no notable distinction between the effects of fluoride varnish and combined fluoride/xylitol varnish on reducing *S. mutans* and *Lactobacillus* colonies in saliva and dental biofilms. However, compared to xylitol varnish, both fluoride and combined varnishes were more successful in decreasing the number of *S. mutans* and *Lactobacillus* colonies in the saliva. Using fluoride and combined fluoride/xylitol varnishes, as shown by their effectiveness in reducing *S. mutans* and *Lactobacillus* colonization, can be a valuable preventive measure to reduce the risk of tooth decay.

## Author Contributions

**Neda Babanouri:** study concept, methodology, project administration, editing the manuscript. **Sarina Sahmeddini:** methodology, writing and editing the manuscript. **Setayesh Khadang:** investigation, methodology, writing the manuscript. **Abdollah Bazargani:** methodology, project administration, editing the manuscript.

## Ethics Statement

The trial was approved by the Ethics Committee of Shiraz University of Medical Sciences (code: IR.SUMS.DENTAl.REC.1400.036) and registered at the Iranian Registry of Clinical Trials (IRCT20181121041713N4). Moreover, based on the principles of the Helsinki (ethical principles of medical research on humans), a comprehensive database of applicable records and information was recorded confidentially and with informed consent.

## Conflicts of Interest

The authors declare no conflicts of interest.

## Data Availability

The data used to support the findings of this study were supplied by the Shiraz University of Medical Sciences under license and so cannot be made freely available. Requests for access to these data should be made to Neda Babanouri (nedababanouri@yahoo.com).
